# Biocompatibility of calcium phosphate bone cement with optimized mechanical properties

**DOI:** 10.1002/jbm.b.33370

**Published:** 2015-03-12

**Authors:** Iwan Palmer, John Nelson, Wolfgang Schatton, Nicholas J. Dunne, Fraser J. Buchanan, Susan A. Clarke

**Affiliations:** ^1^ School of Mechanical and Aerospace EngineeringQueen's University of BelfastBelfastBT9 5AHUK; ^2^ Institute for Global Food Security, School of Biological Sciences, Medical Biology CentreQueen's University of BelfastBelfastBT9 7BLUK; ^3^ KliniPharm GmbHD‐60313 Frankfurt am MainGermany; ^4^ School of Nursing and MidwiferyMedical Biology CentreQueen's University of BelfastBelfastBT9 7BLUK

**Keywords:** calcium phosphate bone cement, collagen, optimized mechanical properties, biocompatibility, bioactivity

## Abstract

The broad aim of this work was to investigate and optimize the properties of calcium phosphate bone cements (CPCs) for use in vertebroplasty to achieve effective primary fixation of spinal fractures. The incorporation of collagen, both bovine and from a marine sponge (*Chondrosia reniformis*), into a CPC was investigated. The biological properties of the CPC and collagen–CPC composites were assessed *in vitro* through the use of human bone marrow stromal cells. Cytotoxicity, proliferation, and osteoblastic differentiation were evaluated using lactate dehydrogenase, PicoGreen, and alkaline phosphatase activity assays, respectively. The addition of both types of collagen resulted in an increase in cytotoxicity, albeit not to a clinically relevant level. Cellular proliferation after 1, 7, and 14 days was unchanged. The osteogenic potential of the CPC was reduced through the addition of bovine collagen but remained unchanged in the case of the marine collagen. These findings, coupled with previous work showing that incorporation of marine collagen in this way can improve the physical properties of CPCs, suggest that such a composite may offer an alternative to CPCs in applications where low setting times and higher mechanical stability are important. © 2015 The Authors. Journal of Biomedical Materials Research Part B: Applied Biomaterials Published by Wiley Periodicals, Inc. 104B:308–315, 2015.

## INTRODUCTION

Fractures of the spine and neck result in over 13,000 annual emergency admissions to hospital in England alone,^1^ with burst fractures accounting for around 15%.^2^ Conservative treatments like bed rest, external bracing and analgesics have been typically used to treat such fractures; however, such treatments are considered by many to be inadequate and are commonly being replaced by minimally invasive surgical procedures.^3^

An example of such a procedure is vertebroplasty—an image‐guided therapy that involves the injection of cement into the vertebral body. It has become a widely used treatment for osteoporotic compression fractures, with up to 84% of patients in some studies experiencing rapid and profound pain relief as well as an increase in functional activities.^4^ Despite this, research into the use of vertebroplasty for the treatment of more severe fractures has been limited and is yet to be realized in a clinical setting.

Poly(methyl methacrylate) (PMMA) is the current cement of choice in vertebroplasty, partly because of its excellent biocompatibility and hemocompatibility.^5^ However, some concerns have been raised regarding its use, such as the high polymerization temperatures, monomer cytotoxicity, and an increased risk of fracture in adjacent vertebral bodies.^6^ Bioactivity can be used to induce positive responses *in vivo*, leading to an interest in calcium phosphate bone cements (CPCs) for this application. CPCs mimic the natural mineral phase of bone and are potentially resorbable, consequently promoting natural bone remodeling and bone ingrowth. CPCs have the potential to be particularly effective in the treatment of burst fractures. The typical mechanisms of injury for such fractures are road traffic accidents and falls from height,^7^ meaning that burst fractures usually occur in the younger population who, in general, have a greater capacity for bone remodeling.^8^ However, concerns remain regarding the use of CPCs in the treatment of burst fractures because of shortcomings in their mechanical properties.^9^^–^ 11

Approximately 30%–35% of the dry weight of bone is organic material, and of this, around 95% is type I collagen.^12^ This collagen plays an important role in bone function and formation, and use of collagen as a biomaterial was investigated as early as the 1970s.^13^ Collagen has been used directly as a biomaterial, in the form of hydrogels, as an additive to calcium phosphate scaffolds, and as an additive to CPCs. It is evident from such work that collagen has the potential to be of significant use in the field of bone regeneration because it has excellent biocompatibility and low antigenicity.^14^ As such, in addition to improving mechanical properties, it is hypothesized that the incorporation of collagen will also result in biological benefits. It has been previously shown that incorporation of bovine collagen into CPCs results in increased cellular adhesion *in vitro* 15, 16; also, *in vivo*, Mai et al.^17^ demonstrated that a bovine collagen–CPC composite was not only biocompatible but also resorbable. Despite this, concerns regarding the clinical safety of collagen remain an obstacle to its use in biomaterials. The clinical incidence of adverse reactions to collagen is extremely rare; the incidence of these reactions is <3%, and they can generally be resolved within a few months.^18^ A much greater concern, particularly because cattle are the primary source of collagen on a commercial scale,^14^ is the risk of transmissible spongiform encephalopathies, such as bovine spongiform encephalopathy. A safer option is to use porcine‐derived collagen, although it has been widely reported that collagen from aquatic and particularly marine sources could be even safer.^14^, ^19^^–^ 22

A possible alternative source of collagen is the common marine Demosponge *Chondrosia reniformis* Nardo, 23). It has been identified worldwide, has a high collagen content,^24^ and a low risk of detrimental toxic compounds.^22^ The fact that *C. reniformis* also reproduces asexually suggests that harvesting of the sponge for collagen isolation could be conducted on a commercial scale.^25^ Swatschek et al.^22^ developed a method for isolating collagen from *C. reniformis* and demonstrated its suitability as a substitute for collagen from conventional sources. The collagen exhibits many features associated with mammalian collagen and, in some aspects, has been shown to display significant similarities to bovine collagen.^26^, ^27^

Such work suggests that using potentially safer collagen from *C. reniformis* in place of bovine‐derived collagen would yield similar benefits to those associated with the use of bovine collagen.

Previous work has shown that a CPC formulated from a powder phase of 100% α‐tricalcium phosphate (α‐TCP‐CPC) displayed superior physical properties to a CPC based on a commercial formulation initially developed by Merck KGaA (Merck‐CPC),^28^ as well as meeting several of the clinical requirements for vertebroplasty.^29^ When compared with Merck‐CPC, the compressive strength of α‐TCP‐CPC was more than doubled from ∼15 to ∼32 MPa, and a near 70% increase in Young's modulus from ∼650 to ∼1100 MPa was observed. These enhancements in mechanical properties were coupled with a threefold improvement in injectability, based on a method developed by Khairoun et al.,^30^ from around 15% to over 50%. In addition, the incorporation of 1 wt % bovine collagen into Merck‐CPC resulted in significant improvements in fracture toughness (*p* < 0.01) and final setting time (*p* < 0.001) without compromising compressive strength, Young's modulus, or injectability. It should be noted, however, that injectability was reduced by around a third.^31^ Furthermore, the incorporation of collagen from the marine Demosponge *C. reniformis* (Nardo, 23) has also been shown to significantly reduce both the initial and final setting times of α‐TCP‐CPC to within the clinical range required for vertebroplasty without compromising its compressive strength, Young's modulus, or injectability.^28^, ^32^^–^ 34

Based on these earlier investigations, the aim of this study was to assess the *in vitro* biological response associated with collagen–CPC composites, from both bovine and marine sponge origin, formulated from α‐TCP‐CPC.

## MATERIALS AND METHODS

### Cement production

α‐TCP‐CPC was formulated from a powder phase of ∼94% α‐TCP (produced in‐house according to Jack et al.^35^) and a liquid phase of 5 wt % Na_2_HPO_4_ in deionized water at a liquid‐to‐powder ratio of 0.35 mL/g. The powder and liquid components were mixed for 1 min, delivered into a polytetrafluoroethylene (PTFE) (RS Components, Northamptonshire, UK) mold within 4 min, and then placed in an oven held at 37 °C for 20 min. The dimensions of the cement samples produced from the PTFE mold were 2 mm in height and 4 mm diameter. The PTFE mold containing the cement was placed in a bath of Ringer's solution for 5 days at 37 °C to ensure complete setting.

Collagen–CPC composites were produced using broadly the same method as earlier. Bovine‐derived collagen (Sigma‐Aldrich, Dorset, UK) and collagen extracted from *C. reniformis* (KliniPharm GmbH, Frankfurt, Germany) were incorporated into α‐TCP‐CPC at a loading of 1 wt % to form composites referred to as BC‐CPC and MC‐CPC, respectively. Prior to incorporation into the powder phase of the cement, the bovine fibers were cryogenically ground using a Freezer Mill 6850 (Rondol Technology, Staffordshire, UK) for two cycles of 2 min, a method previously shown to reduce fiber length.^36^ The dry powder components were turbo blended at 1600 ± 10 rpm for 1 min (DAC 150 FVZ Speed Mixer, Hauschild & Co. KG, Hamm, Germany) to ensure thorough mixing. The marine fibers were suspended in the liquid phase of the cement before mixing with the powder phase.

Two cement formulations were used as controls: Merck‐CPC and a PMMA‐based cement. Merck‐CPC was formulated from a powder phase of 61% α‐TCP, 26% calcium phosphate (CaHPO_4_), 10% calcium carbonate (CaCO_3_), and 3% hydroxyapatite (Plasma Biotal, Derbyshire, UK) and a liquid phase of 4 wt % di‐sodium hydrogen phosphate (Na_2_HPO_4_) (Fisher Scientific, Leicestershire, UK) in deionized water mixed at a liquid‐to‐powder ratio of 0.35 mL/g. It is based on a commercial formulation developed by Merck KGaA (Darmstadt, Germany) to have a similar chemical composition and crystalline structure with that of the mineral phase of bone.^37^ The PMMA cement used was Vertebroplastic Radiopaque Resinous Material (DePuy, Leeds, UK), which is a commercially available cement routinely used clinically for vertebroplasty (referred to herein as VP‐PMMA).

Surface roughness (*R*_a_) of each cement type was assessed using a stylus profilometer (Talysurf 4, Taylor Hobson, Leicester, UK). All samples, barring VP‐PMMA, which was produced under aseptic conditions as per the manufacturer's instructions, were sterilized using γ‐irradiation in accordance with ISO‐11137‐1:2006^38^ (Isotron Applied Sterilisation Technologies, Synergy Health, Swindon, UK). Sterilization using γ‐irradiation has been reported as the most suitable sterilization technique for collagen extracted from *C. reniformis*.^39^

### *In vitro* evaluation

*In vitro* evaluation was carried out using human bone marrow stromal cells (hBMSCs), which are primary cells similar to those that would be in contact with the implant *in vivo* and are capable of differentiating along the osteogenic lineage.^40^

Following informed patient consent, approximately 6 mL of bone marrow was collected from the tibial medullary canal of a 19‐year‐old male undergoing a tibial intramedullary rod fixation procedure at Belfast's Royal Victoria Hospital. The sample was suspended 1:1 in sterile phosphate‐buffered saline (PBS). The resulting suspension was passed through a 21‐gauge needle several times to break up large clumps of bone marrow, before the cells were washed and resuspended in 8 mL of sterile PBS. The mononuclear cell fraction was separated using density centrifugation with Lymphoprep (Axis‐Shield, Dundee, UK) at a speed of 600*g* for 40 min. Separated cells were plated at a seeding density of 1–3 × 10^5^ cells/cm^2^ in minimum essential medium alpha modification with l‐glutamine and without ribonucleosides (Analab, Co., Antrim, UK) supplemented with a further 1% l‐glutamine (Gibco, Invitrogen, Paisley, UK), 1% penicillin–streptomycin solution (Gibco), and 10% foetal bovine serum (FBS GOLD, Analab). Cells were left undisturbed for 1 week and fed twice weekly thereafter. When the cells had been cultured to near confluency, they were passaged using 0.25% trypsin/EDTA (Gibco) at a ratio of 1:4 to expand the cell population.

Cement samples were placed in 96‐well tissue culture plates and seeded with hBMSCs at passage four (P4) at a density of 5 × 10^4^ cells/cm^2^. Outcome measures were cytotoxicity, determined by lactate dehydrogenase (LDH) assay; proliferation, determined by PicoGreen assay; and differentiation, determined by alkaline phosphatase (ALP) activity assay. The LDH and PicoGreen assays were performed with a CytoTox 96 Non‐Radioactive Cytotoxicity Assay kit (Promega, Madison, WI) and a Quant‐iT PicoGreen dsDNA Reagent and Kit (Molecular Probes, Invitrogen), respectively, in accordance with the manufacturer's instructions. The amount of ALP present in the lysates was quantified through the use of a standard curve, produced by adding known amounts of *p*‐nitrophenylphosphate to 0.1% Triton X‐100 in PBS. The assay buffer (1.5*M* alkaline buffer solution) was diluted 1:3 with dH_2_O. A 100‐mg capsule of phosphatase substrate was dissolved in 100 mL of the assay buffer (all from Sigma‐Aldrich). Two hundred microliters of substrate was added to 50 μL of cell lysates and standards in a 96‐well assay plate before being protected from light, and incubated at 37 °C for 30 min. The reaction was stopped by adding 50 μL of 3*M* sodium hydroxide (Sigma‐Aldrich), and absorbance read at 405 nm (GENios Microplate Reader). A PicoGreen assay was performed on the same cell lysates to allow ALP activity to be normalized to cell number.

Cytotoxicity was measured at day 1, and proliferation measured at days 1, 7, and 14. At each time point, there were five repeats of each formulation; four on which the assays would be carried out and an additional one for scanning electron microscopy (SEM) analysis. Cement samples without cells served as a negative control. All wells were filled with 200 μL of complete medium, apart from the day 1 plate, which was filled with phenol red‐free Dulbecco's modified Eagle medium (Gibco) containing supplements as earlier. Cells were fed twice weekly for the duration of the experiment.

Differentiation was measured at days 7, 14, and 21. PicoGreen and ALP activity assays were performed at all three time points to allow ALP activity to be normalized to cell number. In addition to the wells containing samples, cells were added to three empty wells, and all wells were filled with 200 μL of complete medium. When the cells in the three empty wells were near confluency, complete medium was replaced with osteogenic medium (complete medium with additional supplements: 50 μ*M* ascorbate‐2‐phosphate, 0.1 μ*M* dexamethasone, and 10 μ*M* β‐glycerophosphate) (all from Sigma‐Aldrich). Cells were fed twice weekly with osteogenic medium for the duration of the experiment.

SEM was also used to qualitatively assess the biocompatibility of the cements at days 7 and 14. Cells were cultured as described earlier, and specimens were fixed and dried through graded alcohols and hexamethyldisilazane (Sigma‐Aldrich) before being sputter coated with pure gold (Polaron E5150, Polaron Instruments, Doylestown, PA) and viewed using a JEOL JSM 840 (Jeol, Hertfordshire, UK) at an accelerating voltage of 15 kV, and images recorded onto Ilford FP4 film (Ilford Photo, Cheshire, UK) before being scanned.

### Statistical analysis

One‐way analysis of variance (ANOVA) was used to identify significant differences between cement formulations. Where a significant difference was indicated by ANOVA, pair‐wise comparisons to assess intergroup differences were carried out using Tukey's honestly significant difference tests, provided the sample sizes were the same. If sample sizes were not consistent (for example, due to lost or erroneous readings) pair‐wise comparisons were carried out using Gabriel's procedure.

All statistical analyses herein were performed using PASW Statistics 18.0 (IBM Corp., Armonk, NY). A *p*‐value of ≤0.05 was considered significant throughout.

## RESULTS

### Surface roughness

**Figure 1:**
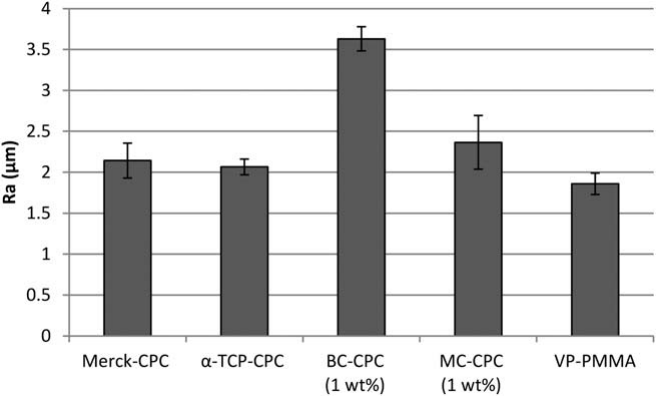
Surface roughness of samples (mean ± standard error; *n* = 4 (*n* = 7 for VP‐PMMA)).

All cements had similar roughnesses, with the exception of BC‐CPC; the other formulations were ∼30%– 45% less rough compared with BC‐CPC (Figure 1).

### Cytotoxicity

**Figure 2:**
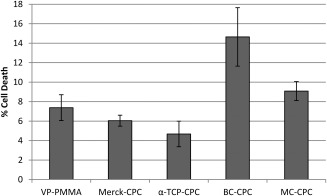
Percentage of total cell death associated with each cement type as assessed with an LDH assay at day 1 (cells seeded at P4; mean ± standard error; *n* = 6 for all cement types except for α‐TCP‐CPC where *n* = 5).

The percentage cell death associated with all cement types was relatively low (<15%) (Figure 2). The lowest percentage cell death was observed on the α‐TCP‐CPC and the highest on BC‐CPC. ANOVA indicated that BC‐CPC induced significantly higher cytotoxicity than all of the other cements, with the exception of MC‐CPC (*p* = 0.038, 0.009, and <0.001 when compared against VP‐PMMA, Merck‐CPC, and α‐TCP‐CPC, respectively). Further, α‐TCP‐CPC was also shown to induce significantly less cytotoxicity compared with MC‐CPC (*p* = 0.022).

### Scanning electron microscopy

**Figure 3:**
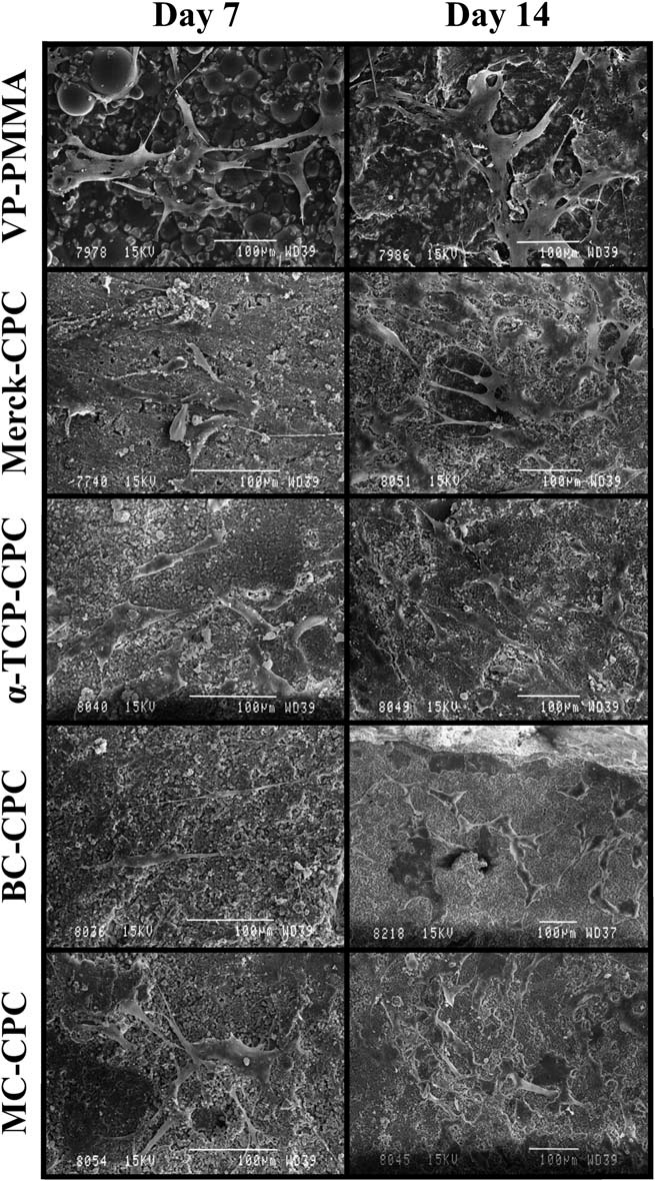
Cell growth on the cements at days 7 and 14.

SEM confirmed cell attachment and qualitatively demonstrated proliferation (Figure 3). All cement formulations supported cell attachment and proliferation with many filopodia extending from cells to the cement surface and to adjacent cells. Qualitatively, an increase in cell number was observed over time. No clear distinction was evident between the CaP‐based formulations, although BC‐CPC and MC‐CPC seemed to support cell growth equally well.

### Proliferation

**Figure 4:**
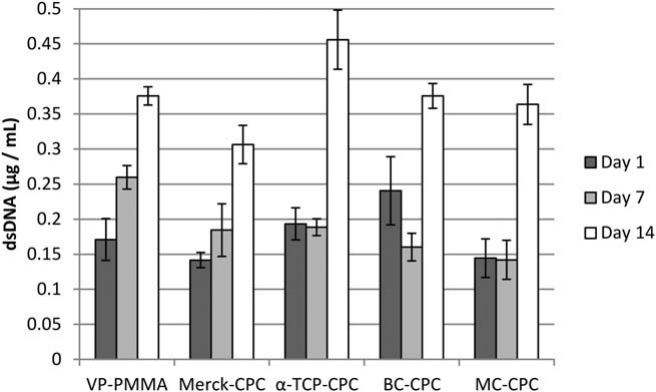
Cell number on the cements based on the amount of dsDNA recorded following a PicoGreen assay (cells seeded at P4; mean ± standard error; *n* = 5).

There was a general increase in cell number with time, indicating that all cement formulations support cell proliferation (Figure 4). The amount of dsDNA more than doubled on all cement formulations over the 14‐day culture period. No significant differences between the cements were observed at day 1 (*p* = 0.171). At day 7, there were significantly more cells on the VP‐PMMA cement than on MC‐CPC (*p* = 0.021), but this was no longer evident at day 14. Instead, there was now a significantly higher cell number on α‐TCP‐CPC when compared with Merck‐CPC (*p* = 0.008).

### Differentiation

**Figure 5:**
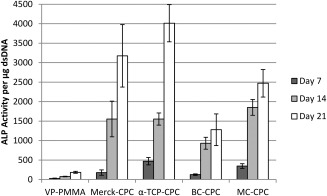
ALP activity per microgram dsDNA for all cement formulations (cells seeded at P4; mean ± standard error; *n* = 5, apart from Merck‐CPC at day 14 where *n* = 4).

Large increases in ALP activity were observed as the experiment progressed (Figure 5). The largest increases were seen between days 7 and 14, where increases in ALP activity of over 160% were observed on all cement formulations and both Merck‐CPC and BC‐CPC, demonstrating increases well in excess of 500%. This trend continued from day 14 to day 21, though the increases in ALP activity were less marked, typically ranging from around 30% to 160%.

There was an effect of cement formulation on ALP activity. Cells cultured on VP‐PMMA cement showed a lower ALP activity when compared with the CaP‐based formulations, and this was statistically significant at each of the time points (Table 1). Cells cultured on BC‐CPC had a lower ALP activity compared with that of the other CaP‐based formulations at all time points, and this was significantly different than the unaugmented α‐TCP‐CPC at days 7 (*p* = 0.046) and 21 (*p* = 0.002). Merck‐CPC supported significantly lower ALP activity compared with that of α‐TCP‐CPC at day 7 (*p* = 0.038), but no significant differences were observed at the latter two time points.

**Table 1:** Statistical Analyses Comparing ALP Activity for All Cement Types at Each Time Point as well as Each Time Point for All Cement Types

Time Point	Cement Comparison		*p*‐Value
Day 7	VP‐PMMA	Merck‐CPC	0.015*
		α‐TCP‐CPC	<0.001*
		BC‐CPC	0.012*
		MC‐CPC	<0.001*
	Merck‐CPC	α‐TCP‐CPC	0.038*
		BC‐CPC	>0.999
		MC‐CPC	0.144
	α‐TCP‐CPC	BC‐CPC	0.046*
		MC‐CPC	0.958
	BC‐CPC	MC‐CPC	0.17
Day 14	VP‐PMMA	Merck‐CPC	<0.001*
		α‐TCP‐CPC	<0.001*
		BC‐CPC	<0.001*
		MC‐CPC	<0.001*
	Merck‐CPC	α‐TCP‐CPC	0.999
		BC‐CPC	0.684
		MC‐CPC	0.876
	α‐TCP‐CPC	BC‐CPC	0.243
		MC‐CPC	0.997
	BC‐CPC	MC‐CPC	0.059
Day 21	VP‐PMMA	Merck‐CPC	<0.001*
		α‐TCP‐CPC	<0.001*
		BC‐CPC	<0.001*
		MC‐CPC	<0.001*
	Merck‐CPC	α‐TCP‐CPC	0.798
		BC‐CPC	0.02*
		MC‐CPC	0.96
	α‐TCP‐CPC	BC‐CPC	0.002*
		MC‐CPC	0.408
	BC‐CPC	MC‐CPC	0.081

*Denotes a significant difference.

## DISCUSSION

The novel α‐TCP‐CPC, which has been optimized for its mechanical and handling properties, was compared against Merck‐CPC, a relevant market comparator. In addition to displaying superior physical properties, this study confirms that α‐TCP‐CPC maintains a similar level of biocompatibility and bioactivity to Merck‐CPC. No significant difference in cytotoxicity was observed while proliferation was significantly higher on α‐TCP‐CPC; beyond the first time point, the level of differentiation was also retained in the novel formulation.

The percentage cell death (3%–7%) reported for the unaugmented CPCs is in line with previously published data for tissue culture plastic and lower than that reported for other biomaterials commonly used in this field, for example, hydroxyapatite.^41^ The incorporation of collagen resulted in a slight increase in cytotoxicity. This is in contrast to some published work which suggests that incorporation of bovine collagen into CPCs results in increased cellular adhesion.^15^, ^16^ Reasons for this difference are unclear; however, differences in both CPC formulation and collagen handling, in addition to cell type, may be responsible. The increased cell death observed on BC‐CPC may be a result of an increased surface roughness, which has been shown to lower surface adhesion of osteoblasts.^42^ This was not the case with the MC‐CPC, which has a surface roughness not significantly different to that of the α‐TCP‐CPC and VP‐PMMA cements because of the preparation methods.^43^ Therefore, a possible explanation for the slight increase in cytotoxicity with this augmented cement is that, despite the seemingly low risk of toxic compounds from *C. reniformis* as an edible sponge,^44^ some detrimental compounds are retained in the collagen after the extraction process. Although extracts from the sponge had no significant deterrent effects on fish^45^ and crabs,^46^ work by Sepčić et al.^47^ has shown that an aqueous extract from *C. reniformis* homogenate displays some cytocidal activity as well as low haemolytic activity. Despite this, the fact that both collagen‐augmented CPCs displayed a lower level of cytotoxicity than is associated with other commonly used biomaterials suggests that the level of cytotoxicity associated with the collagen‐augmented CPCs is not likely to be clinically relevant. This was confirmed by SEM analysis, which showed cells adhering and adopting the flattened and elongated morphology associated with hBMSCs^48^ on all cement formulations.

Proliferation was quantified using a PicoGreen assay. There was a general trend toward increasing cell number for all cements, with a significant increase in cell number measured between days 7 and 14. However, no significant variation was seen between day 1 and day 7 for any of the cements, with the exception of VP‐PMMA, where the increase in dsDNA concentration was more gradual with significant increases seen between each of the three time points. It is unclear why proliferation seems to be delayed for the CPC systems compared with the VP‐PMMA, although it may be as a result of the increased differentiation, which is known to result in the down‐regulation of proliferation.^49^ It has also been suggested that the initial seeding of cells onto CPCs can result in altered adhesion characteristics, thus affecting cell growth.^50^ It is surmised that this change in adhesion characteristics is partially due to the acidification of the culture media. Further, the reduction of available divalent cations as a result of calcium‐deficient materials has also been shown to compromise cell adherence,^51^ which may also explain the observed delay in proliferation. Despite this, cells generally proliferated equally well on all of the cement types.

In addition to proliferation, the degree of osteogenic differentiation was also assessed by quantifying ALP activity. Differentiation was observed on all cement types and was shown to increase with time. Increased osteogenic potential was displayed by cells cultured on all of the CPC systems when compared with VP‐PMMA, a finding which was expected due to the lack of bioactivity associated with PMMA.

It is possible that factors other than cement composition are responsible for these differences. However, identifying such factors remains difficult because the mechanism underlying osteoinduction by biomaterials is not fully understood.^52^ Barradas et al.^52^ have summarized some factors involved in osteoinduction; these include, in addition to chemical composition, implant geometry, porosity, surface roughness, and specific surface area. It has also been shown that nanopatterns on the surface of PMMA can induce mesenchymal stem cells to differentiate along the osteogenic lineage, even in the absence of osteogenic supplements.^53^

The differing levels of ALP activity displayed by BC‐CPC compared with Merck‐CPC and α‐TCP‐CPC may be a result of the differences in surface topography. The surface roughness of BC‐CPC was significantly higher than both that of MC‐CPC and α‐TCP‐CPC.^43^ The effect of topography on cell behavior is complex and needs to be considered on multiple scales,^54^ with topography on the nanoscale shown to affect differentiation.^53^ However, it has been suggested that differentiation can be delayed by rougher surfaces,^48^ which is in agreement with the findings reported in this study.

Cells cultured on BC‐CPC had lower osteogenic capacity compared with those on MC‐CPC, and this may be due to differences in the collagens used. To the best of the authors' knowledge, this work is the first investigation comparing the biocompatibility of collagen from *C. reniformis* with that derived from bovine Achilles tendon. Indeed, few studies have compared the biocompatibility of marine collagens with that of mammals. However, in a study comparing scaffolds of jellyfish collagen with those produced from type I collagen from calf skin, the jellyfish collagen did not induce a significant cytotoxic effect on human fibroblasts and supported higher cell viability compared with the bovine collagen.^14^ Although no significant difference in cytotoxicity was observed between the marine and bovine collagens evaluated herein, the work involving the jellyfish collagen does highlight the potential for interspecific differences in the *in vitro* behavior of collagens.

In a study by Nagai et al.,^55^ human periodontal ligament fibroblasts (HPdLFs) were cultured on cross‐linked salmon collagen gel and porcine collagen gel. HPdLF growth rate was shown to be faster on the salmon gel when compared with that on the porcine gel. Also, HPdLFs cultured on the salmon gel displayed a higher level of ALP activity than that of those cultured on the porcine gel, as well as an increased messenger RNA expression of type I collagen and osteocalcin. The use of collagen gels rather than fibers coupled with the fact that different species were used limits the comparisons that can be made between the study by Nagai et al. and the findings presented in this study. Notwithstanding this fact, it is interesting to note that ALP activity was higher for the fish‐derived collagen gel in comparison with the mammalian‐derived collagen gel. This is in agreement with the finding that ALP activity was higher in MC‐CPC compared with BC‐CPC; although it can only be speculated that these similarities are a result of the aquatic source of the collagens involved.

## CONCLUSIONS

This study demonstrated that all of the CPCs considered supported cell attachment and improved osteogenicity when compared with VP‐PMMA.

α‐TCP‐CPC displayed comparable *in vitro* biological properties with Merck‐CPC. Also, despite demonstrating a significant, but clinically irrelevant increase in cytotoxicity, the incorporation of 1 wt % collagen derived from *C. reniformis* had an insignificant influence on the *in vitro* biological properties of α‐TCP‐CPC. This finding, coupled with the knowledge that incorporation of marine collagen in this way improves the physical properties of α‐TCP‐CPC, suggests that it may be a suitable alternative to some of the clinically used bone cements in application such as vertebroplasty, where mechanical stability is essential.
